# Network pharmacology: an efficient but underutilized approach in oral, head and neck cancer therapy—a review

**DOI:** 10.3389/fphar.2024.1410942

**Published:** 2024-07-05

**Authors:** Pandiyan Muthuramalingam, Rajendran Jeyasri, Venkatramanan Varadharajan, Arumugam Priya, Anand Raj Dhanapal, Hyunsuk Shin, Muthu Thiruvengadam, Manikandan Ramesh, Murugesan Krishnan, Rebecca Oziohu Omosimua, Divyan Devasir Sathyaseelan, Baskar Venkidasamy

**Affiliations:** ^1^ Division of Horticultural Science, College of Agriculture and Life Sciences, Gyeongsang National University, Jinju, Republic of Korea; ^2^ Department of Biotechnology, Science Campus, Alagappa University, Karaikudi, India; ^3^ Department of Biotechnology, PSG College of Technology, Coimbatore, India; ^4^ Department of Medicine, Division of Gastroenterology and Hepatology, Pennsylvania State University College of Medicine, Hershey, PA, United States; ^5^ Chemistry and Bioprospecting Division, Institute of Forest Genetics and Tree Breeding (IFGTB), Coimbatore, India; ^6^ Department of Crop Science, College of Sanghuh Life Science, Konkuk University, Seoul, Republic of Korea; ^7^ Department of Oral and Maxillofacial Surgery, Saveetha Dental College and Hospitals, Saveetha Institute of Medical and Technical Sciences (SIMATS), Saveetha University, Chennai, India; ^8^ Nigerian Institute of Medical Research, Lagos, Nigeria; ^9^ Department of General Surgery, Saveetha Medical College and Hospitals, Saveetha Institute of Medical and Technical Sciences (SIMATS), Saveetha University, Chennai, India

**Keywords:** oral cancer, HNC, database, network pharmacology, bioactives, therapy

## Abstract

The application of network pharmacology (NP) has advanced our understanding of the complex molecular mechanisms underlying diseases, including neck, head, and oral cancers, as well as thyroid carcinoma. This review aimed to explore the therapeutic potential of natural network pharmacology using compounds and traditional Chinese medicines for combating these malignancies. NP serves as a pivotal tool that provides a comprehensive view of the interactions among compounds, genes, and diseases, thereby contributing to the advancement of disease treatment and management. In parallel, this review discusses the significance of publicly accessible databases in the identification of oral, head, and neck cancer-specific genes. These databases, including those for head and neck oral cancer, head and neck cancer, oral cancer, and genomic variants of oral cancer, offer valuable insights into the genes, miRNAs, drugs, and genetic variations associated with these cancers. They serve as indispensable resources for researchers, clinicians, and drug developers, contributing to the pursuit of precision medicine and improved treatment of these challenging malignancies. In summary, advancements in NP could improve the globalization and modernization of traditional medicines and prognostic targets as well as aid in the development of innovative drugs. Furthermore, this review will be an eye-opener for researchers working on drug development from traditional medicines by applying NP approaches.

## 1 Introduction

**FIGURE 1:**
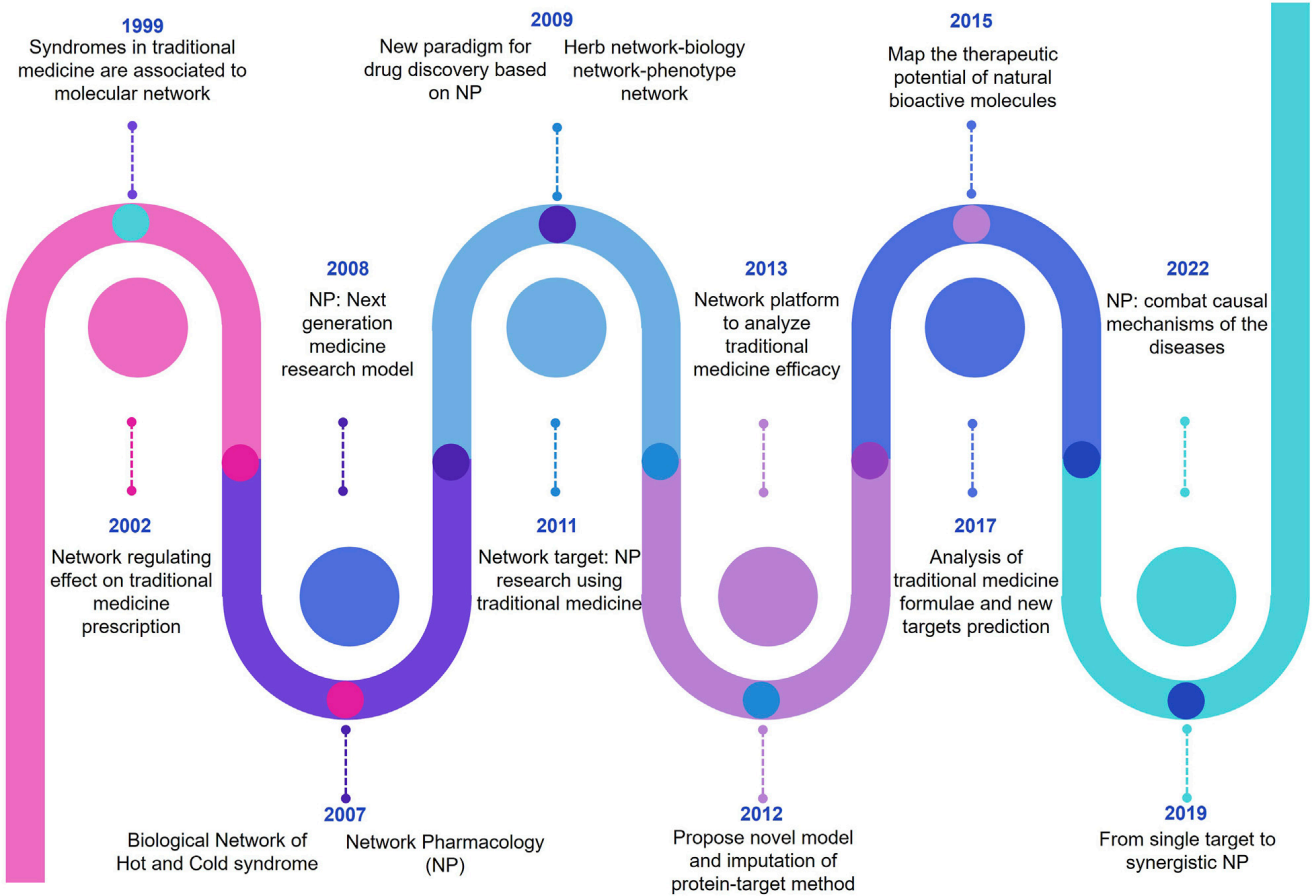
Pictorial representation of evolutionary timelines of network pharmacology (NP).

Network pharmacology (NP) is an innovative research approach that integrates concepts from pharmacology, bioinformatics, and network analysis to understand complex interactions within biological systems and advance drug discovery. It focuses on comprehensively mapping the relationships between drugs, genes, proteins, and diseases in a network framework. In this methodology, data from various sources, including biological databases, clinical studies, and experimental results, are systematically analyzed to identify potential drug targets, understand drug mechanisms of action, and uncover novel therapeutic interventions (Muthuramalingam et al., 2023a; Zhao et al., 2023). In recent days, NP is emerging as a pioneer in modern era drug discovery and development. This is due to its unique feature to combine the information science and systematic medicine. In addition, Figure 1 represents the timelines of NP.

Head and neck cancer (HNC), a prevalent and combative malignancy with a high mortality rate (Johnson et al., 2020). It originates from various primary sites, including the oral cavity, oropharynx, nasopharynx, larynx, hypopharynx, and tongue, with squamous cell carcinoma (SCC) being predominant. Distinct types of head and neck squamous cell carcinoma (HNSCC) include oral squamous cell carcinoma (OSCC), oropharyngeal squamous cell carcinoma (OPSCC), laryngeal squamous cell carcinoma (LSCC), and hypopharyngeal squamous cell carcinoma (HSCC) (Mao et al., 2004). OSCC is a major histopathological class of oral cancer. The major factors contributing to HNC include genetic diversity, alcohol consumption, tobacco, Epstein–Barr virus, and human papillomavirus (HPV). Standard treatments include radiation therapy, chemotherapy, surgery, and chemoradiotherapy. Recent advances in molecular-targeted therapy and immunotherapy offer hope for advanced-stage patients. However, outcomes remain suboptimal, necessitating the discovery of novel prognostic markers and therapeutics (Ferraguti et al., 2022).

HNSCC is a major global health concern, with limited early identification and prognostic markers. Diagnosis often occurs at later stages, resulting in a 40%–50% 5-year survival rate with conventional treatments. Recurrent HNSCC is associated with a normal survival of just one year (Li et al., 2023). A previous study indicated that MYO1B might affect tumorigenesis and prognosis by modulating the immune microenvironment of HNSCC (Li et al., 2023). This study uncovered a competing endogenous RNA (ceRNA) network associated with CCND1 in HNSCC, identifying the TPRG1-AS1-hsa-miR-363-3P-MYO1B pathway as a potential diagnostic marker and therapeutic target, and shed light on the molecular mechanisms underlying HNSCC progression and prognosis (Li et al., 2023). The relationship between lymphatic metastasis and CEACAM5, including its possible regulatory role in HNSCC, suggests its potential as a prognostic marker and therapeutic target for HNSCC (Wang et al., 2022). In addition, other genes, such as CEACAM6, play a major role in tumor growth, and chemotherapeutic sensitivity has been assessed both *in vivo* and *in vitro* (Cameron et al., 2012). Furthermore, Wang et al. (2024) reported a direct correlation between the expression levels of the CDKN2A gene and patient outcomes. CDKN2A gene is a crucial prognostic marker in HNSCC, serving to establish enhanced and personalized therapeutic targets and also involved in cell cycle progression. Database analysis revealed that the mRNA level of chloride channel accessory 4 (CLCA4) was frequently lower in primary tumor tissues than in non-cancerous colon tissues and even lower in liver metastases. Wei et al. (2020) revealed that the expression levels of CLCA4 mRNA are associated with lower overall survival rates in patients with colorectal cancer (CRC), breast cancer, HNC, and stomach cancer. Overall, downregulation of CLCA4 expression may influence the development and progression of CRC. Conventional treatments can lead to severe complications and side effects, which further challenge patient wellbeing. Traditional Chinese medicine (TCM) has shown promise in HNSCC treatment, with herbs such as Poriacocos, *Atractylodes macrocephalakoidz*, and *Artemisia escopariaeherba* effectively managing complications. YinchenWuling San (YWLS), comprising of six herbal ingredients, is another potential alternative for HNSCC therapy. However, the active components and antitumor mechanisms of YWLS require further investigation (Zhang et al., 2022).

OSCC is a malignancy that does not obtain effective treatment. Chenpi, an easily accessible TCM, has been explored for its potential anticancer properties by using NP. Tangeretin, a compound found in Chenpi, is a key active ingredient in OSCC treatment as indicated by the component-target-disease network. Core genes such as PIK3R1, ESR1, and CDK1 were identified through protein-protein interaction and survival analysis. Molecular docking predicts the binding of compounds to their targets. Tangeretin inhibited cell growth by cell cycle arrest in the S phase, apoptosis induction, and regulation of core transcription in SCC25 cells, with less toxicity to HOK cells (Yu et al., 2022).

**FIGURE 2:**
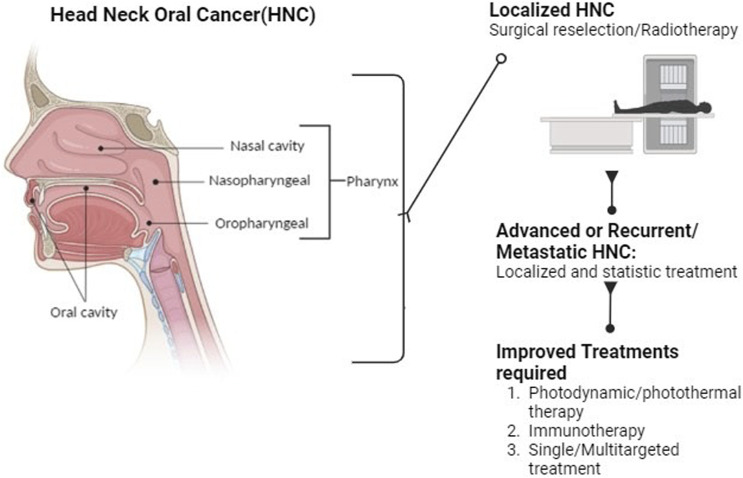
Diagrammatic representation of Oral, Head and neck cancer and their treatment modalities.

Recent insights and prospects in combating cisplatin resistance in HNSCC are complex, and the primary treatment for advanced HNSCC involves cisplatin-based concurrent radiotherapy (Figure 2). Unfortunately, cisplatin resistance is a significant problem that can lead to poor treatment outcomes. To combat this resistance, it is essential to understand its underlying mechanisms. Cisplatin resistance in HNSCC involves multiple factors, including cancer stem cells, epithelial-mesenchymal transition, autophagy, drug expulsion, and metabolic changes. Recent developments in nanodrug delivery methods, small-molecule suppressors, and genetic technologies offer new opportunities for modulating cisplatin resistance in HNSCC (Yu et al., 2022). Natural molecules could be an ideal approach to attenuate drug-resistant cancers, including oral and HNC cancers (Krishnan and Venkidasamy, 2022; Rekha et al., 2022; Ling et al., 2023).

NP are a comprehensive method for identifying potential clinical drug targets. It involves molecular docking, a key technique for predicting target-drug interactions (Huang et al., 2023). By integrating these two approaches, a more precise reference for the use of clinical drugs and repurposing of effective therapeutics can be obtained. Thus, it serves as an effective supplementary method for identifying metformin furoate targets and the underlying mechanisms in the fight against HNSCC (Panossian and Effeth, 2022).

It is interesting that NP approaches in cancer therapy are highly beneficial, as they deviate from the conventional discovery of drugs by labelling the drug’s ability to target numerous protein pathways involved (Li et al., 2023). In addition, several non-cancer-related genes/proteins representing unusual drug targets have been identified through NP as potential candidates for the pharmacological treatment of oral and HNC cancer.

This holistic review first outlines the potential perspectives of NP in the treatment of less-studied oral, HNC. Furthermore, the databases utilized for identification of oral, HNC related genes, tools used in NP, cell signaling pathways related to oral cancer, and the comprehensive methodology in NP were discussed in detail.

## 2 Databases for identification of oral, HNC specific genes

The Cancer Database is a comprehensive collection of data pertaining to different types of human cancers at the genetic and molecular levels. This valuable resource is utilized for a deeper understanding of the various stages of cancer development, and to facilitate advancements in cancer treatment (Tang et al., 2022). HN and oral cancers are major and widespread cancers, with a survival rate of approximately 50% over 5 years. Among all cancer types, HNC and oral cancer families may exhibit a large range of variations (Johnson et al., 2020). OSCC is a common cancer among patients with HNSCCs. Current scientific investigations in this field have focused on the etiology and therapy of cancer. Modern genomics technologies, such as transcriptomics, microarrays, gene sequencing, and proteomics, have generated vast amounts of data (Supplitt et al., 2021; Dai and Shen, 2022). These studies identified numerous differentially expressed genes and miRNAs, and the resulting information has been deposited in numerous databases. Extensive data derived from diverse sources have emphasized the necessity of collaboration and data sharing to effectively utilize this newfound knowledge. This part of review offers comprehensive details regarding publicly accessible databases that comprise relevant data regarding OSCC and HNSCC. These databases allow us to determine the oral and HNC associated targets and their molecular mechanisms and assist in drug repurposing as well as novel drug development.

### 2.1 Head and neck oral cancer database (HNOCDB)

HNOCDB is a specialized dataset that offers comprehensive details regarding the different types of HNSCCs at the molecular level. HNOCDB (http://gyanxet.com/hno.html) is publicly available to academic and nonprofit users (Mitra et al., 2012). This database encompasses various HNSCCs cases, including OSCC, and provides detailed insights into their genes and miRNAs. The information contained in the database was obtained from experimental evidence and extracted from textual sources. The HNOCDB data are stored under three primary links: genes, miRNAs, and chromosomes. The links between genes and miRNAs consist of sublinks related to different types of carcinoma subtypes of HN cancer, including oral, salivary, tongue, laryngeal, thyroid, and pharyngeal cancers. Each sub-link contained data on genes and miRNAs for each subtype. The link “Chromosomes” contains human chromosomes contain a human chromosome map. Each chromosome was arranged separately in terms of its genes and miRNAs. This database contains data on 133 genes, including information on the mechanisms underlying oncogene and miRNA activation, physical location of genes/miRNAs on chromosomes, gene- and miRNA-related mutations, and biochemical properties of gene/miRNA products.

### 2.2 Head and neck cancer database (HNCDB)

HNCDB (http://hncdb.cancerbio.info) was created by combining information from the scientific literature obtained through text mining of PubMed abstracts with manual curation to gather solid data on genes and drugs related to HNC. Gene expression data were obtained from GEO and TCGA databases. The HNCDB has three interconnected components: “HNC GENE,” “Connectivity Map,” and “ANALYSIS.” The “HNC GENE” section provides detailed findings on 1,173 genes joined with HNC, which were curated manually from 2,564 publications. The “Connectivity Map” comprises data on the potential links between 176 drugs, curated from 2,032 publications, and the 1,173 genes allied to HNC. The “ANALYSIS” component enables users to perform differential expression, correlation, and survival assessment on the 2,403 samples obtained from 78 gene expression results focused on HNC (Zhang D. et al., 2019). Altogether, HNCDB will be of great interest to patients with HNC and will enhance the development of precision medicine research in the field of HNC.

### 2.3 Oral cancer gene database (OrCGDB)

The OrCGDB (http://www.tumor-gene.org/Oral/oral.html) was created primarily to provide the biomedical community with a smooth tool to obtain the most recent data on genes associated with oral cancer. The location of each gene on the chromosome, mutation type, oncogenic activation mechanisms, biochemical characteristics, and function were specified for each gene in this database, which has been organized according to its name. In addition, the database includes gene synonyms and clinical information related to each gene. Currently, OSCC has been linked to 15 genes and 1367 associated factors, and the total number is likely to be higher (Levine and Steffen, 2001). To facilitate the search process, users can explore the OrCGDB by entering either a gene name or a word text. This comprehensive database also ensures that each piece of information is supported by corresponding MEDLINE citations.

### 2.4 Oral cancer database (OrCa-dB)

OrCa-dB serves as a comprehensive resource that contains information on genes, miRNAs, proteins, pathways, and distinct expression patterns related to the development and progression of oral cancer. It was created through meticulous manual curation of PubMed records, paying specific attention to the genes affected by molecular and genetic occurrences during the evolution of oral cancer. It comprises clinically pertinent details, namely, the influence of socioeconomic status on early diagnosis, the assets of screening interventions, the use of chemotherapy and targeted therapy, ongoing clinical trials in oral cancer, patient management, and considerations for their quality of life, and is a standard feature of OrCa-dB. Each entry in OrCa-dB is associated with external databases, including SwissProt, PDB, UniProt, miRBase, KEGG pathway database, and EMBL, providing the additional benefit of accessing relevant biological knowledge (Reshmi et al., 2012).

### 2.5 Database of GENomic variants of oral cancer (dbGENVOC)

A comprehensive open-access website called dbGENVOC (https://research.nibmg.ac.in/dbcares/dbgenvoc/) enables users to investigate and analyze different types of genetic variations (such as somatic and rare germline single-nucleotide variants), insertions, and deletions discovered through whole exome and genome sequencing in patients with oral cancer in India. With curated and regularly updated gene-level summary statistics, dbGENVOC serves as a valuable resource for researchers, enabling them to perform correlation analyses, diagnostic tests, and wet-lab validations on significant targets. Access to population-specific cancer-associated variants made available by dbGENVOC also makes it easier to comprehend the sub-phenotypes and functional consequences of oral cancer. This repository is useful to researchers seeking insightful data in this field (Pradhan et al., 2021).

Recent advancements in genome sequencing and relevant technologies have contributed to the integration of scientific research and therapeutic applications through the development of a database of easily available data and analytical software. Notably, only a few databases specifically exist for HNSCC and OSCC. This is only one of the sections of most databases. Certain complications hinder the creation of specific databases that include all the genes involved in OSCC, the heterogeneity of cancer cells, and inconsistent responses to antigens among patients with identical symptoms. To address this problem, advanced computational and statistical approaches focused on large-scale data management and mining should be established to attain the clinical significance of cancer genome discovery.

### 2.6 Databases and tools used in network pharmacology

NP is a novel method for examining mechanisms and advancements in drug development (Noor et al., 2022; Muthuramalingam et al., 2023b). Several databases and tools have attributed prime support to NP research (see Table 1). Commonly used databases for NP research include Traditional Chinese medicine (TCM) databases, such as TCMdatabase@Taiwan (Chen, 2011), TCMGeneDIT (Fang et al., 2008), TCMSP (Ru et al., 2014), HIT (Ye et al., 2010), and TCMID (Xue et al., 2013). Additionally, compound and drug detail databases such as PubChem, Drugbank (Wishart et al., 2018), ChEMBL (Gaulton et al., 2012), and STITCH (Kuhn et al., 2014), target interaction databases such as Reactome (Matthews et al., 2009), MINT (Zanzoni et al., 2002), STRING (Szklarczyk et al., 2015), HAPPI (Chen et al., 2009), HPRD (Peri et al., 2003), and IntAct (Kerrien et al., 2012), and gene-disease-related databases including OMIM (Hamosh et al., 2005) and GAD (Becker et al., 2004) play a significant role. These biological databases and the results of clinical trials can be used by researchers to examine the network of interactions between herbal substances, proteins/genes, and diseases. Through this system, the pharmacology perspective allows for a deeper understanding of how herbs affect diseases. Moreover, the mining of valuable information from databases is significantly facilitated by the NP algorithms and tools. One commonly used algorithm is the Random Walk (Chen et al., 2012), which clusters networks by measuring the similarity between a randomly selected node (drug, target, or disease) and its adjacent nodes. Another algorithm, PRINCE (Vanunu et al., 2010), helps prioritize disease genes and establish associations between protein complexes through restrictions on the sorted function that emphasizes network smoothness and prior knowledge. Despite data collection and analysis, visualization plays a crucial role in NP, making the networks more easily comprehensible. Cytoscape is a freely available platform that is well-suited for visualizing networks of molecular interactions and biological pathways (Shannon et al., 2003). It also allows for the integration of these networks with annotations, transcription profiles, and other relevant data. Pajek (Dohleman, 2006) is another robust tool used to analyze complex nonlinear networks. These easily accessible databases and tools empower researchers to identify prognostic genes, compound targets, target-associated mechanisms, and retrieve other valuable information from the viewpoint of NP research. In addition, these are useful for employing the NP approach as a modern drug discovery process. Moreover, a comprehensive understanding of signaling pathways is essential to fully utilize these databases and tools for identifying the potential drug targets.

**TABLE 1:** List of databases and tools used for network pharmacology.

S. No	Databases/Tools	Description	Pros	Cons	Website	Reference
TCM databases						
1	TCMID (Traditional Chinese Medicine Integrated Database)	It connects the gap between TCM and contemporary life sciences contains details about TCM formulas and compounds of herbs	• Serve as a comprehensive repository • Facilitates pathway and network analysis of TCM ingredients and their target proteins • Support for drug discovery and development	• The accuracy and completeness of data in TCMID may vary depending on the availability and quality of input data • TCMID must adhere to ethical principles and regulatory requirements	http://www.megabionet.org/tcmid/	Xue et al. (2013)
2	TCMSP (Traditional Chinese medicine systems pharmacology database)	Tool that captures the connections between drugs, targets and diseases	• Facilitates the identification of bioactive compounds • Assists in target identification and validation • Provides mechanistic insights into the therapeutic effects of TCM • Enables predictive modeling and network analysis	• Limitations of predictive models • TCMSP analyses may oversimplify the biological complexity and context sensitivity	http://lsp.nwu.edu.cn/tcmsp.php	Ru et al. (2014)
3	TCMGeneDIT (Database of traditional Chinese medicine, gene, and disease information using text mining)	Information about association between TCMs, genes, diseases, TCM effects and TCM ingredients	• Integration of diverse data sources • Employs text mining and natural language processing techniques • Easy accessibility and user-friendly interface	• Incomplete Coverage and Bias • Text mining algorithms may struggle with semantic ambiguity and context sensitivity	http://tcm.lifescience.ntu.edu.tw/	Fang et al. (2008)
4	HIT 2.0 (Herbal Ingredients’ Targets Database Introduction)	HIT is a manually curated database that provides details on the protein targets of compounds found in Chinese herbs	• Available to the scientific community and promoting collaboration	• It may have limitations in terms of the completeness and accuracy of the data • It may require specialized knowledge and skills to effectively navigate and interpret the data	http://hit2.badd-cao.net	Yan et al. (2022)
5	TCM@Taiwan (Database of traditional Chinese medicine @Taiwan)	Currently the globes hugest non-commercial TCM database. It provides information about 61,000 compounds comprised in 453 TCM herbs taken from Chinese medical texts and scientific articles	• Supports evidence based practice • Provides comprehensive information on TCM	• Limited Coverage of Clinical Trials • Requires regular updates and maintenance	http://tcm.cmu.edu.tw/	Chen (2011)
6	NPACT (Naturally occurring Plant based Anticancerous Compound-activity-Target database)	The NPACT database, compiled from 762 articles, contains chemicals obtained from plants that exhibit anti-cancerous activity	• Facilitates drug discovery efforts by identifying potential anticancer compounds • Integrates data from multiple sources • Covers a diverse array of chemical structures and compounds • User-Friendly Interface	• The database may provide a simplified representation of anticancer mechanisms, requiring additional experimental validation and mechanistic studies for comprehensive understanding	http://crdd.osdd.net/raghava/npact/	Mangal et al. (2013)
7	CancerHSP (Anticancer Herbs database of Systems Pharmacology)	It records anticancer herbal medicines with anticancer molecules. It shows the molecular structure and nine key ADME features of each molecule	• Facilitates Drug Discovery • Offers a holistic view of the pharmacological properties of anticancer herbs, helping researchers to identify potential therapeutic targets and mechanisms of action	• It requires continual updates and maintenance to incorporate new data, improve data quality, and address software bugs or compatibility issues	http://lsp.nwsuaf.edu.cn/CancerHSP.php	Tao et al. (2015)
8	NPASS (Natural Product Activity and Species Source Database)	It governs comprehensive details of species sources and biological functions of natural products	• The database includes data on the species sources of natural products, allowing researchers to identify the organisms from which specific compounds are derived • Facilitates Drug Discovery and functional food development	• Data limitations • Complexity of biological activities • Validation challenges	http://bidd2.nus.edu.sg/NPASS/	Zeng et al. (2018)
DRUG TARGET DATABASES						
9	DrugBank	DrugBank attributes detailed molecular information on drugs and their mechanisms, comprising details on their chemical, pharmacological, pharmaceutical, and ADME, interactions and their targets	• Easy to access • Regularly updated with new information on drugs\ • It offers powerful search functionality	• Access to certain advanced features or datasets may require a subscription fee • Limited coverage • Lack of clinical evidence	http://www.drugbank.ca/	Wishart et al., 2006; Wishart et al., 2018
10	TTD (Therapeutic target database)	It gives details on drugs, targets, targeted diseases, and pathways	• TTD provides tools for target prioritization • Integration with other databases • Comprehensive Information • TTD is freely accessible online	• The quality of data in TTD may vary • The data may be incomplete or limited for certain targets	http://bidd.nus.edu.sg/group/ttd/ttd.asp	Li et al. (2018)
11	STITCH (Search Tool for Interacting Chemicals)	It is a database of known and predicted interactions between chemicals and proteins	• Users can access a vast amount of chemical information • Many search tools can integrate data from various sources • Some tools offer visualization features • Advanced search tools may incorporate predictive modeling algorithms	• Integrating data from disparate sources can be complex, leading to compatibility issues or incomplete datasets • Access to high-quality chemical databases may come with a significant cost	http://stitch.embl.de/	Kuhn et al. (2014)
11	STITCH (Search Tool for Interacting Chemicals)	It is a database of known and predicted interactions between chemicals and proteins	• Users can access a vast amount of chemical information • Many search tools can integrate data from various sources • Some tools offer visualization features • Advanced search tools may incorporate predictive modeling algorithms	• Integrating data from disparate sources can be complex, leading to compatibility issues or incomplete datasets • Access to high-quality chemical databases may come with a significant cost	http://stitch.embl.de/	Kuhn et al. (2014)
12	ChEMBL	It composes more drug-like bioactive compounds than other drug target databases	• Open access • It contains a vast amount of data on bioactive molecules • High-quality data • Provides detailed structural information on bioactive molecules	• ChEMBL strives to provide high-quality data, errors, inconsistencies, or outdated information may still exist in the database • Limited experimental details	https://www.ebi.ac.uk/chembldb	Gaulton et al. (2012)
13	ZINC	It attributes details about ligands and their purchasability, targets, clinical trials, etc.	• Large compound library • Diverse chemical space • Easy to access • Integration with virtual screening tools • Offers customization options for compound selection	• Limited structural information • The quality control of compounds in the ZINC database may vary • Access to the ZINC database is free, the cost of purchasing compounds for experimental validation can be significant	http://zinc15.docking.org	Sterling and Irwin (2015)
14	BindingDB	It governs experimentally calculated protein– ligand binding affinity data	• Vast data repository • Offers a user-friendly interface • Provides structural information for many protein-ligand complexes, including protein crystal structures and ligand conformations • Free access	• Complexity of analysis • Lack of structural coverage	www.bindingdb.org	Gilson et al. (2016)
WEB SERVERS FOR TARGET PREDICTION						
15	SuperPred	A web tool for predicting targets and the ATC (Anatomical Therapeutic Chemical) codes of molecules	• Predict potential interactions between drugs and protein targets • Provides access to a large library of compounds • User-friendly interface • Rapid predictions of drug-target interactions	• Operates as a black-box model, meaning that users have limited visibility into the underlying algorithms and features used for prediction • Limited compounds coverage	http://prediction.charite.de	Nickel et al. (2014)
16	TargetNet	It predicts the targets of molecules based on the QSAR model, which mathematically communes precise chemical traits of molecules to their bioactivities	• High-throughput screening • Can predict interactions between various types of molecules • It offers a cost-effective and time-efficient approach	• TargetNet may produce false positives and false negatives • Limited scope of prediction	http://targetnet.scbdd.com	Yao et al. (2016)
17	SwissTargetPrediction	Web tool to identify the targets of compounds based on a combo of 2D and 3D similarity computes with known compounds	• Large target database • User-friendly interface • Fast prediction • Integration with other tools	• Users have limited visibility into the underlying algorithms and features used for prediction	http://www.swisstargetprediction.ch	Gfeller et al. (2014)
18	SystemsDock	SystemsDock evaluates the potential of protein–ligand binding and presents the potential with a score using a molecular docking algorithm	• User-Friendly Interface • Comprehensive analysis • Integration of multiple data types	• Integrating diverse types of biological data can be challenging due to differences in data formats, quality, and reliability	http://systemsdock.unit.oist.jp/	Hsin et al. (2016)
19	PharmMapper	It predicts protein targets of compounds based on pharmacophore model	• Supports high-throughput target prediction • Enables users to predict potential target proteins for small molecules based on pharmacophore mapping	• It primarily focuses on predicting interactions between small molecules and individual target proteins • Limited predictive accuracy	http://lilab.ecust.edu.cn/pharmmapper/	Wang et al. (2017)
DISEASE DATABASES						
20	OMIM (Online Mendelian Inheritance in Man database)	The curated database OMIM offers details on the genetic basis and the related genes for all known Mendelian disorders	• It is considered an authoritative source of information on genetic disorders • User-friendly interface with powerful search functionality • Provides links to relevant scientific literature • Contains curated information on the relationships between genes and diseases	• Primarily focuses on monogenic disorders with a well-defined genetic basis, which may limit its coverage of complex diseases with multifactorial or polygenic inheritance patterns	http://www.ncbi.nlm.nih.gov/omim/	Hamosh et al. (2005)
21	MalaCards	Human disease database which is an integrated compilation of human diseases and their annotations	• Facilitates the exploration of gene-disease associations • User-friendly interface • Regularly updated with new research findings and data	• Access to certain features or advanced functionalities of MalaCards may require a subscription or payment	http://www.malacards.org/	Rappaport et al. (2017)
22	DisGeNET	The detailed gene-disease association (GDA) database that attributes the current knowledge of human genetic diseases	• Multiple evidence types • Employs semantic integration techniques to harmonize data from disparate sources and standardize gene and disease annotations	• Limited annotation details • It contains a wealth of data on gene-disease associations, which can be complex and challenging to analyze	http://www.disgenet.org	Pinero et al. (2016)
23	DigSee	A text mining search engine which governs evidence sentences exploring genes participated in the emergence of disease via biological processes	• Focus on protein-protein interactions • Includes information on how protein-protein interactions are implicated in diseases	• The accuracy of information in DigSee can vary • Access to certain advanced functionalities of DigSee may require a subscription or payment	http://gcancer.org/digsee	Kim et al. (2013)
WEB SERVERS AND TOOLS FOR NETWORK AND FUNCTIONAL ANALYSIS						
24	Cytoscape	A free tool for combining, visualizing, and examining data in the context of networks	• Provides a wide range of visualization options • Offers a comprehensive suite of network analysis tools, enabling users to perform various network analyses • Integration with bioinformatics databases • Cytoscape features a plugin architecture • It has a large and active user community	• Lack of native analysis tools • Users may encounter compatibility issues or bugs when using certain plugins	http://www.cytoscape.org/	Shannon et al. (2003)
25	BATMAN-TCM (Bioinformatics Analysis Tool for Molecular mechANism of TCM)	The online bioinformatics tool for studying the molecular mechanism of TCM.	• Facilitates the identification of biological pathways and processes associated with TCM compounds • Employs computational algorithms to predict potential interactions between TCM compounds and target proteins	• The accuracy and reliability of BATMAN-TCM predictions depend on the availability and quality of the input data	http://bionet.ncpsb.org/batman-tcm	Liu et al. (2016)
26	DAVID (Database for Annotation, Visualization and Integrated Discovery)	It governs enrichment analysis for the gene list from diverse biological facets	• Provides comprehensive functional annotation tools • It offers enrichment analysis tools • DAVID offers customizable analysis options	• DAVID has limitations on the size of the input gene • DAVID relies on predefined functional annotations and gene sets from existing databases	http://david.abcc.ncifcrf.gov	Huang et al. (2009)
27	CMap (Connectivity Map)	Online tool for finding disease–gene–drug association based on the similarity of their gene-expression signatures	• Large-scale data • Open Access • CMap facilitates the identification of the molecular mechanisms of action for bioactive small molecules • CMap enables drug repurposing efforts by identifying existing drugs or compounds	• Gene expression profiles in CMap are generated from cultured human cell lines, which may not fully recapitulate the complexity of *in vivo* biological systems or tissue-specific responses to small molecule treatments • Limited mechanistic insights	https://clue.io	Lamb et al. (2006); Subramanian et al. (2017)

## 3 Cell signaling pathways involved in head and neck cancer

HNSCC includes cancers that are initiated in the squamous cells of the inner mucosa of the mouth and throat, thereby involving the oral cavity, sinuses, nasal passage, pharynx, and larynx (Arantes et al., 2014; Johnson et al., 2020). Although a wide range of histologies is known, squamous cell carcinoma (SCC) is the most frequently observed type. Ranking sixth globally in terms of high mortality rate and poor diagnosis, this type of cancer is linked to environmental- and lifestyle-associated predisposing risk factors, such as tobacco usage, alcohol intake, and HPV infection (GBD 2016 Disease and Injury Incidence and Prevalence Collaborators, 2017; Auguste et al., 2020; Chauhan et al., 2022).

Disruption of cellular homeostasis by impaired DNA repair and cell cycle regulation diminishes the potential for normal carcinogen metabolism. Individuals with inherited genetic compositions that metabolize carcinogenic agents are more susceptible to HNSCC (Scully et al., 2000). Therapeutic regimens including chemotherapy, radiation therapy, surgery, and molecular-based treatments are available to increase patient survival. For several HNSCC cases, a single therapeutic regimen is insufficient, and a multimodal approach combining chemotherapy, radiation, surgery, and monoclonal antibody (MAb) treatment is required (Bossi et al., 2016). The 5- years overall survival of HNSCC patients remains poor despite the development of advanced therapeutics (Cho et al., 2018). As invasive surgery and radical therapy provide a relatively low survival rate, research focus has shifted to the development of specific molecular-targeting agents. Cetuximab, an MAb against EGFR, is the only targeted therapy approved by the FDA for HNSCC. The discovery of immunotherapy with programmed death receptor-1 (PD-1) blocking antibodies such as nivolumab and pembrolizumab has extended the therapeutic window for HNSCC treatment (Mehra et al., 2008; Wise-Draper et al., 2022).

Expanding our understanding of the molecular mechanisms and cellular pathways involved in the pathogenesis and histology of HNSCC will assist in the development of novel molecular therapeutics by altering the pathways and downstream signaling effectors. Eventually, identification of targeted molecular therapy not only increased the treatment options or survival rate of the patients but also reduced the toxicity to normal cells by selectively killing the cancer cells. To develop such a targeted molecule, one should understand an ideal target that is only specific to tumorous cells and commonly found in all cancerous cells. Ideal molecular targets can be identified with a better understanding of signaling pathways. Therefore, the development of modern approaches to target specific signaling pathways and their associated biological networks underlying specific diseases is a prerequisite. In this part of the review, the molecular signaling pathways that commonly participate in HNSCC are discussed in detail.

### 3.1 Epidermal growth factor receptor (EGFR) pathway

The first and foremost molecular target that has been demonstrated to improve HNSCC is EGFR, which belongs to the ErbB family of receptor tyrosine kinase (RTK). Several studies have elucidated the crucial role of EGFR in HNSCC. Disruption of ErbB family member receptors has been shown to play a central role in the regulation of various cellular activities, such as cell division, differentiation, and migration (Schlessinger, 2004). Moreover, altered EGFR expression has been linked to other human cancers, including breast cancer, glioblastoma, and lung cancer. In the inactive state, EGFR is auto-inhibited and found in the plasma membrane. Ligand binding induces EGFR autophosphorylation and conformational changes leading to receptor activation (Kovacs et al., 2015). This ligand-dependent EGFR induction activates multiple signaling pathways, including the PI3K/AKT, Ras/MAPK, phospholipase C (PLC)/protein kinase C (PKC) signaling cascade, and the activation of transcription factors (TFs), thereby regulating crucial cellular events, such as survival, proliferation, differentiation, and migration (Lemmon and Schlessinger, 2010). Since more than 90% of patients with HNSCC have overexpressed EGFR or random mutations, targeting this receptor is crucial for impeding cancer progression (Kalyankrishna and Grandis, 2006; Nair et al., 2022). Alterations in the expression of EGFR or mutations in this gene influence overall and progression-free survival (Johnson et al., 2020). Studies have reported that the normal mucosa of patients with HNSCC has increased EGFR expression compared to that of healthy individuals. Additionally, based on tumor progression from hyperplasia to invasive carcinoma, a gradual increase in EGFR expression has been observed (Grandis and Tweardy, 1993; Grandis et al., 1996; Zimmermann et al., 2006). Although overexpression of EGFR is commonly observed in HNSCC, other cytogenetic and molecular alterations, including the expression levels of proteins and their activation, aberrant gene copy number (GCN), mutations, polymorphisms, and expression of EGFRvIII and ligands, also play important roles in HNSCC (Bossi et al., 2016). Disease-free survival in patients expressing EGFR alterations has been observed to be far lower than that in other (Hama et al., 2009). These characteristic features of EGFR in HNSCC are predictors of responsiveness to chemotherapy, radiation, and survival percentage (Nair et al., 2022). In addition to pre-existing alterations and mutations in EGFR, tumors tend to develop resistance by obtaining mutations in regions where therapeutic antibodies bind. Several mutations have been suggested in antibody-specific EGFR target domains, such as extracellular ligand-binding and tyrosine kinase domains (Cerami et al., 2012; Gao et al., 2013). Overexpression of EGFR has been linked with radiation resistance, as EGFR repairs radiation-mediated double-strand breaks by forming an EGFR-DNA-dependent protein kinase (PK) complex (Dittmann et al., 2005). Targeting EFGR using anti-EGFR (monoclonal antibodies mAbs) or inhibitors of the kinase domain along with radiation therapy has become a standard therapeutic strategy for HNSCC (Sundvall et al., 2010; Fasano et al., 2021).

Cetuximab (CTX), an anti-EGFR chimeric human-murine monoclonal antibody, has been widely utilized in HNSCC therapy; to date, it remains the only approved targeted therapy (Tathineni et al., 2023). CTX binds to the extracellular domain of EGFR by competing with the natural ligands EGF and TGF-α, thereby blocking the downstream cell growth and proliferation signaling cascade. Because CTX has a higher affinity than natural ligands, intracellular domain activation is blocked, which in turn maintains the tyrosine kinase-dependent signal transduction pathway under control (Huang et al., 2002). Blockage of EGFR by CTX also leads to the internalization of EGFR, thereby reducing the availability of receptors on the cell surface. Direct inhibition of the receptor has shown favorable effects in radiation therapy and combination therapy with other chemotherapeutic drugs (Sung et al., 2005; Muraro et al., 2021). Although targeted therapy with CTX has several advantages, CTX treatment slowly leads to acquired resistance in most HNSCC patients for one of the following reasons: alteration in EGFR or downstream effectors, activation of a different pathway or protein involved in tumor progression, and induction of mechanisms involved in the immunosuppressive tumor microenvironment (Chen et al., 2010; Ortiz-Cuaran et al., 2021). The primary reason for the development of resistance against CTX is mutations in genes such as KRAS, NRAS, and EGFR (Allegra et al., 2016). The combination of CTX with other available therapeutic methods, such as chemotherapy, radiation therapy, and their combinations, could meet the current needs. Furthermore, more research and clinical studies should be conducted to improve the efficacy of CTX and to develop new anti-EGFR biologics.

### 3.2 PI3K/AKT/mTOR pathway

Signal transduction through the PI3K/AKT/mTOR pathway significantly influences various cellular processes, including cell growth, motility, differentiation, proliferation, survival, nutrient uptake, and metabolism (Yu and Cui, 2016). The PI3K/AKT/mTOR pathway is one of the most frequently dysregulated pathways in HNSCC patients. Whole-exome sequencing data from 151 HNSCC tumor samples demonstrated that 31% of the tumors had mutations in the PI3K oncogenic pathway (Lui et al., 2013). Mutations in the genes involved in this pathway are the second leading cause of human cancers (Liu et al., 2009). The three major molecules involved in this pathway are protein kinase B (AKT), Phosphatidylinositol-4,5-Bisphosphate 3-Kinase Catalytic Subunit Alpha (PIK3CA), and mammalian target of rapamycin (mTOR) (Dubot et al., 2018). Once activated, PI3K phosphorylates secondary messengers such as phosphatidylinositol, PIP2, and PIP3, and activates the effector molecules of AKT and mTOR. PI3K/AKT/mTOR pathway activation and signal transduction leads to the inactivation of tumor suppressor phosphatase and tensin homolog gene (PTEN), mutation or overexpression of genes including PI3KCA, AKT, and MTOR, and activates various growth factor receptors (Courtney et al., 2010); common alterations in this pathway that are found in HNSCC include reduced expression of PTEN and amplification of AKT (Vander Broek et al., 2013). The heterogeneous nature of HNSCC and various genetic alterations and dysregulation have led to the development of resistance to general and targeted treatments. PI3K pathway activation is resistant to chemotherapy, radiation therapy, and targeted therapeutic molecules including EGFR inhibitors. CTX-resistant tumors have shown increased gene expression and protein activation of the PI3K/AKT pathway compared with CTX-sensitive tumors (Keysar et al., 2013). Thus, overcoming resistance to CTX can lead to improved response and survival rates, and decrease the toxicity induced by radiation and chemotherapy. Therefore, the PI3K/AKT/mTOR pathway should be targeted from a clinical research perspective. Various inhibitors of PI3K (buparlisib, PX-866, and BYL719), mTOR (temsirolimus, Everolimus, Sirolimus), and AKT (MK2206, Panitumumab) are currently under different clinical trial phases, either alone or in combination with other treatments. Knowledge of the mutational alterations in genes that participate in the PI3K/AKT/mTOR pathway and the development of specific inhibitors will enable personalized treatment in the future, which could also increase the response and survival rates.

### 3.3 WNT/NOTCH signaling pathway

Dysregulation of the WNT signaling pathway, either through mutation or altered expression of pathway components, has implications for the invasive development of HNSCC tumors [100]. The role of Wnt in HNSCC was first shown 2 decades before the levels of Fzd and Dvl were observed to be higher in HNSCC tumors than in normal tissues (Leethanakul et al., 2000). Another recent study showed that abnormal induction of the Wnt signaling pathway accelerates tumor transformation in head and neck tissues (Iwai et al., 2005). Various Wnt ligands have diverse roles in HNSCC. Wnt ligands Wnt1, Wnt3, and Wnt3a can promote invasion, inhibit apoptosis, cause dysplasia, and cause metastasis, respectively, through the canonical Wnt signaling pathway (Rhee et al., 2002; Ishida et al., 2007; Zhang Q. et al., 2019). Wnt ligands Wnt4, Wnt5a, and Wnt5b have been shown to enhance migration and invasion, leading to tumorigenesis and metastasis through non-canonical Wnt signaling (Qin et al., 2015; Prgomet et al., 2017). Other Wnt ligands, including Wnt7a, Wnt7b, Wnt10b, and Wnt11, have also been shown to promote cell migration, proliferation, and invasion (Shiah et al., 2014; Xie et al., 2020). β-catenin, the key effector molecule responsible for triggering the transcription of Wnt-specific genes, has also been shown to be linked with HNSCC. A recent study showed that β-catenin overexpression is linked to elevated transcriptional activity in HNSCC (Kartha et al., 2018). Although Wnt/β-catenin signaling pathway-associated mutations are not primarily involved in HNSCC, studies have shown that cross-talk between other signaling pathways, such as FAT1 and AJUBA, can change the activity of the Wnt signaling pathway (Beck and Golemis, 2016). Understanding the signaling pathways that govern disease prognosis is crucial for combating this cancer. The NP approach involves uncovering signaling pathways, bioactives-disease-related target genes, and constructing target gene/protein interaction networks.

## 4 Plant bioactives targeting oral, HNC

**FIGURE 3:**
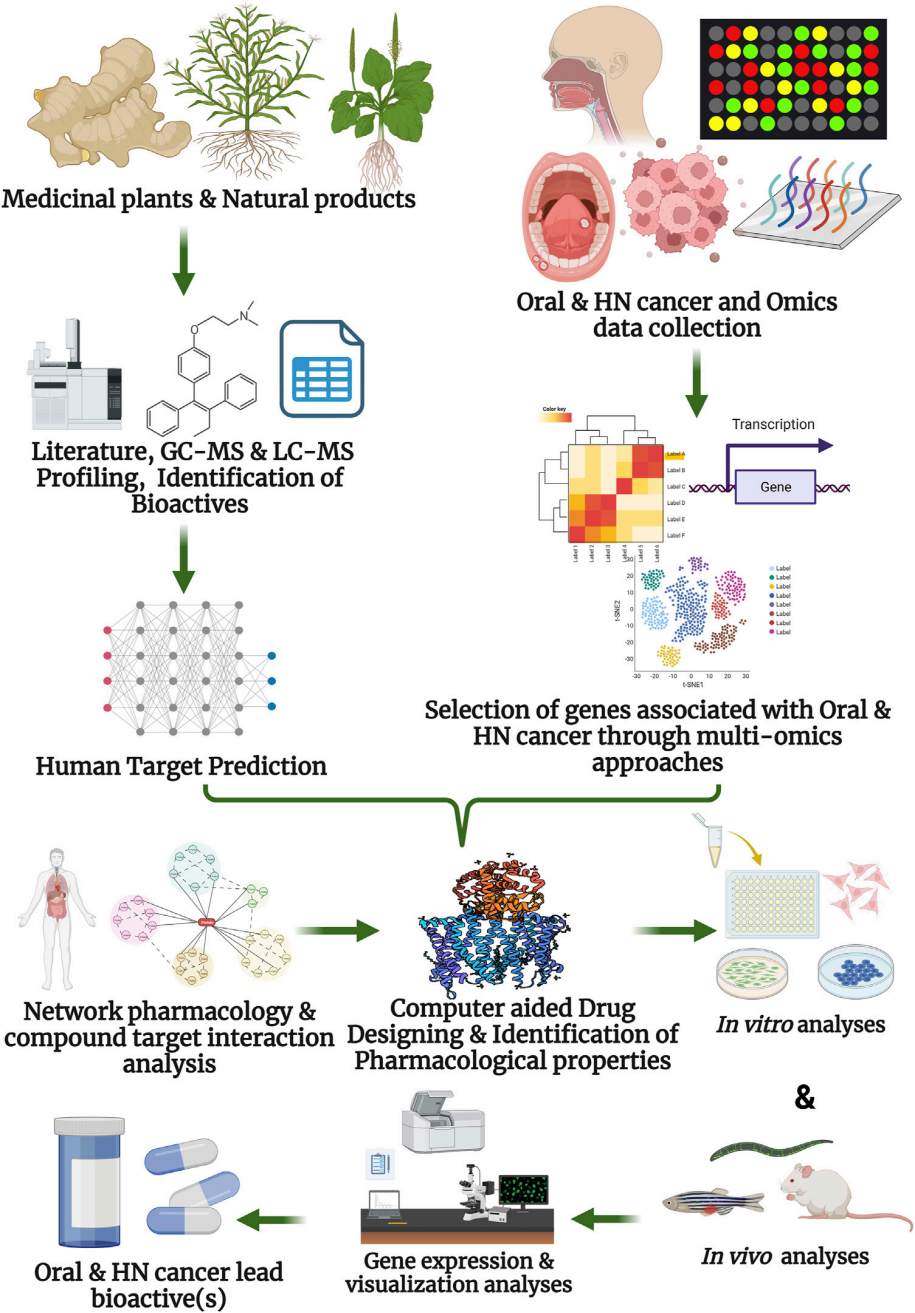
Network pharmacology for elucidation of the anti-cancer activity of plant bioactive compounds against oral, and head and neck cancer.

Plant-derived phytochemicals have shown significant anticancer activity against many types of cancer, including oral and HN cancer. Many of these bioactive compounds from plants are already employed as chemotherapeutic agents in cancer treatment, such as taxanes (from the bark of *Taxus brevifolia*), vinca alkaloids (from *Catharanthus roseus*), and Camptothecin and its derivatives (from *Camptotheca acuminata*), among others (Changxing et al., 2020). Integrating traditional knowledge with modern drug discovery approaches, including NP, has enabled researchers to predict the anticancer properties of several phytocompounds traditionally used in different medicinal systems, such as Sideha, Ayurveda, and traditional Chinese medicine (Halder and Jha, 2023). This approach shows promise in discovering novel therapeutic targets and in understanding the mechanisms of action of plant bioactive compounds, potentially leading to more effective and targeted treatments for oral and HNC (Figure 3). Hence, understanding these plant bioactive molecules and their direct human disease targets using NP is a recent trend in the modern era. In this section, we delineate the plant bioactives and their direct oral HN cancer-associated targets. This will serve as a significant reference for the treatment of oral and HN cancers and further research.

### 4.1 Gene targets of plant bioactives against oral cancer

In different medicinal systems, plant-based remedies, especially formulations, concoctions, or pills, are regularly used to treat oral cancer. Some important plants used to treat oral cancer include neem (*Azadirachta indica*), turmeric (*Curcuma longa*), ginseng (*Panax ginseng*), holy basil (*Ocimum tenuiflorum*), licorice (*Glycyrrhiza glabra*), Indian frankincense (*Boswellia serrata*), aswagandha (*Withania somnifera*), cat claws (*Uncaria tomentosa*), and milk thistle (*Silybum marianum*) (Busia, 2016; Hari, 2020). The anti-inflammatory activity of some of these plants may aid in controlling the inflammation and pain associated with oral cancer. Scientific research on the anticancer properties of certain plants highlights their major phytochemical compounds and mechanisms of action against oral cancer. Notably, curcumin, an active constituent of turmeric, has been shown to effectively impede cancer proliferation and metastasis by disrupting EGFR signaling pathways, particularly by blocking EGFR phosphorylation (Somarriva et al., 2016).

Furthermore, Zhen et al. (2014) found that curcumin inhibits various gene targets associated with OSCC proliferation, including uPAR, uPA, MMP-9 (matrix metalloproteinase-9), and MMP-2. Additionally, we observed the downregulation of genes linked to EGFR signaling, such as STAT3, ERK1/2, and Akt, when the human OSCC cell line (SCC-25) was treated with curcumin, and this effect occurred in a dose-dependent manner. In the case of milk thistle, silymarin (a mixture of silychristin, silibinin, and silydianin) has demonstrated potent anticancer activity. Following the administration of silymarin, human oral cancer cell lines (HSC-4, YD15, and Ca9.22) exhibited increased expression of DR5 and cleaved caspase-8 proteins, resulting in the death of cancer cells via BH3 interacting-domain death agonist (BID) protein cleavage and subsequent TRAIL-induced apoptosis (Won et al., 2018). Moreover, the presence of silymarin led to upregulation of cytochrome c protein expression in oral cancer cells. Consequently, this upregulation causes a reduction in the mitochondrial membrane potential and results in reduced ATP generation. A comprehensive list of phytochemicals, their sources, and modes of action against oral cancer is provided in Table 2.

**TABLE 2:** Phytochemicals and their targets against oral cancer.

Bioactive compounds	Plant source	Target genes	Mechanism of action	Reference
Lycopene	Red fruits and vegetables like watermelons, tomatoes, apricots, pink guavas, and pink grapefruits	*IGF1, IGFBP-1, IGFBP-3* and *FOXO1*	Reduction in cell proliferation by impeding the *IGF1* pathway	Tao et al. (2021)
Isothiocyanate	Broccoli, watercress, brussels sprouts, cabbage	*ATM, CHK2,* and *p53*	Induces G2/M phase arrest and apoptosis, demonstrating notable selectivity with less toxicity towards normal cells	Yeh et al. (2016)
Anthocyanins and other phenolics from berries	Black raspberries	activator protein 1 (AP-1) and *NF-κB*	The extracts display potent anti-proliferative properties against human cancer cells, promoting apoptosis. Furthermore, they effectively inhibit *AP-1* and *NF-κB* in cell cultures	Chatelain et al. (2011)
Garcinol	*Garcinia indica* fruit peel and leaves	*STAT-3, P65, Ki-67* and *CD31*	Effectively inhibits HNSCC cell growth in mice with minimal toxicity. Leads to the downregulation of STAT-3, p65, Ki-67, and CD31	Schobert and Biersack (2019)
Resveratrol	Grapes, apples, blueberries, plums, and peanut	*STAT-3*	Resveratrol effectively suppresses the STAT3 cascade in HNSCC cells by inducing Suppressors of cytokine signaling (SOCS)-1. This induction results in the inhibition of *STAT3* phosphorylation and subsequently hinders cell proliferation	Baek et al. (2016)
Catechins	*Camellia sinensis*	*P21, P53* and *P73*	Effectively disrupts *P21, P53,* and *P73*, leading to cell cycle arrest and enhanced apoptosis, thereby inhibiting cancer cell proliferation	Ramshankar and Krishnamurthy, (2014)

The application of NP has facilitated the discovery of several gene targets linked to various plant compounds with known anti-oral cancer properties. For example, Yu et al. (2022) identified specific anti-oral cancer targets of tangeritin, a bioactive constituent derived from aged citrous peels. Citrous peel is renowned in TCM owing to its potential anticancer properties. Tangeritin targets *CDK1*, *ESR1*, and *PIK3R1*. *In vitro* analysis using the SCC 25 cell line demonstrated upregulation of all core targets, as predicted by the NP approach. Another study by Ladke et al. (2022) showed that NP can be used to identify anti-oral cancer gene targets of bioactive compounds present in *G. glabra*. They discovered that the bioactive compound wortmannin possesses anti-oral cancer properties by targeting the hub gene androgen receptor and disrupting the PI3K-AKT pathway (Ladke et al., 2022).

Similarly, Zhang et al. (2021) employed the NP approach to elucidate the anticancer mechanism of Huanglianjiedu decoction against oral cancer. The authors identified approximately 35 bioactive compounds in the decoction, with 52 hub genes associated with these compounds. The central mechanism responsible for the anticancer effect was attributed to apoptosis induced by the inhibition of the phosphorylation of NF-κB p65 and ERK1/2 (Zhang et al., 2021). These studies collectively underscore the utility of NP, combined with *in vitro* investigations, in pinpointing the core targets of plant bioactive compounds and uncovering the mechanisms underlying their anti-oral cancer properties.

### 4.2 Gene targets of plant bioactives against HNC

Plants such as tea (*Camellia sinensis*), turmeric (*C. longa*), mistletoe (*Viscum album*), and aloe vera (*Aloe barbadensis* miller) have traditionally been used in medicinal systems to treat HNC (Sagar et al., 2006; Al-Achi, 2020). Current research on natural anticancer compounds has shed light on the distinctive mechanisms of action found in these plants. For example, curcumin targets inhibitor kappa B kinase (IκK) and triggers apoptosis in HNSCC by suppressing the NF-κB pathway (Wilken et al., 2011). In contrast, catechins, particularly epigallocatechin-3-gallate (EGCG) from the tea plant, hinder the metastasis of HNSCC cells by focusing on the RECK (Reversion Inducing Cysteine Rich Protein with Kazal Motifs) gene. RECK is a negative regulator of MMP, and decreased expression of MMP-2 and MMP-9 is associated with reduced invasion and metastasis of cancer cells (Kim and Kim, 2014). Table 3 presents a comprehensive list of the targets of the bioactive compounds derived from various plants. A notable observation from this table is that the majority of these bioactive target signaling pathways are closely linked to cell apoptosis.

**TABLE 3:** Phytochemicals and their targets against HNC.

Bioactive compound	Plant source	Target genes	Mechanism of action	Reference
Guggulsterone	*Commiphora mukul*	Phosphotyrosine and *STAT-3*	Inhibition of *STAT* -3	Leeman-Neillet al. (2009)
Bitter melon extract	Bitter Melon	*MCL1, c-MYC* and *p-STAT-3*	Inhibits cell proliferation by targeting c-met signaling	Rajamoorthi et al. (2013)
Lycopene	Red fruits and vegetables such as watermelons, tomatoes, apricots, pink guavas, and pink grapefruits	*MMP-2, MMP-9* and *VEGF*	Stops invasion and metastasis by inhibiting *VEGF* mediated angiogenesis	Irani (2020)
Resveratrol	Grapes, apples, blueberries, plums, and peanut	*MEK/ERK/NF-kB*	Induces Apoptosis	Shrotriya et al. (2014)
Luteolin	Broccoli, thyme, pepper, celery	*NF-κB*	Blocks the *NF-κB* pathway	Majumdar et al. (2014)
Isothiocyanate	Broccoli, watercress, Brussels sprouts, cabbage	*EGFR, IKK* and *IκB*	Suppressed EGF-stimulated SAS cell proliferation by inactivating *NF-κB* through phosphorylated *IKK* and *IκB*, which resulted in the reduction of *MMP-2* and *MMP-9* expression	Chen et al. (2013)

NP has significantly enhanced our understanding of the gene targets linked to traditional formulations used for treating HNC, shedding light on the underlying mechanisms of action. For example, Mi et al., 2020 conducted an NP-based analysis of LeiGongTeng (Tripterygii Radix), a Chinese remedy for cancer, and found that its active constituents (beta-sitosterol, nobiletin, and kaempferol) exert anticancer effects by targeting the RELA, ESR1, CASP3, and VEGFA genes. Similarly, Gao et al., 2021 investigated the gene targets of Baiying Qinghou decoction in laryngeal SCC (LSCC). The essential anticancer targets associated with the decoction were identified as TP53, NOS3, IL1B, and EGFR. In both studies, the PI3K-Akt signaling pathway was recognized as a crucial pathway influenced by bioactive compounds present in these remedies. Moreover, an NP study of Yinchen Wuling San, a complex medical prescription comprising six medicinal plants used for cancer treatment, revealed that the genes TNF, AKT1, and EGFR are targeted by the bioactive compounds of Yinchen Wuling San against HNSCC (Zhang et al., 2022). The gene targets obtained through NP could be further validated through molecular docking and other *in silico* experiments to confirm the effects of bioactive compounds on these targets. In the future, more investigations are required to uncover the multi-target pharmacological mechanism of plant bioactives against oral, HN cancer.

## 5 *In silico* confirmation of anticancer effect of bioactives against oral, neck and head cancer using docking

**FIGURE 4:**
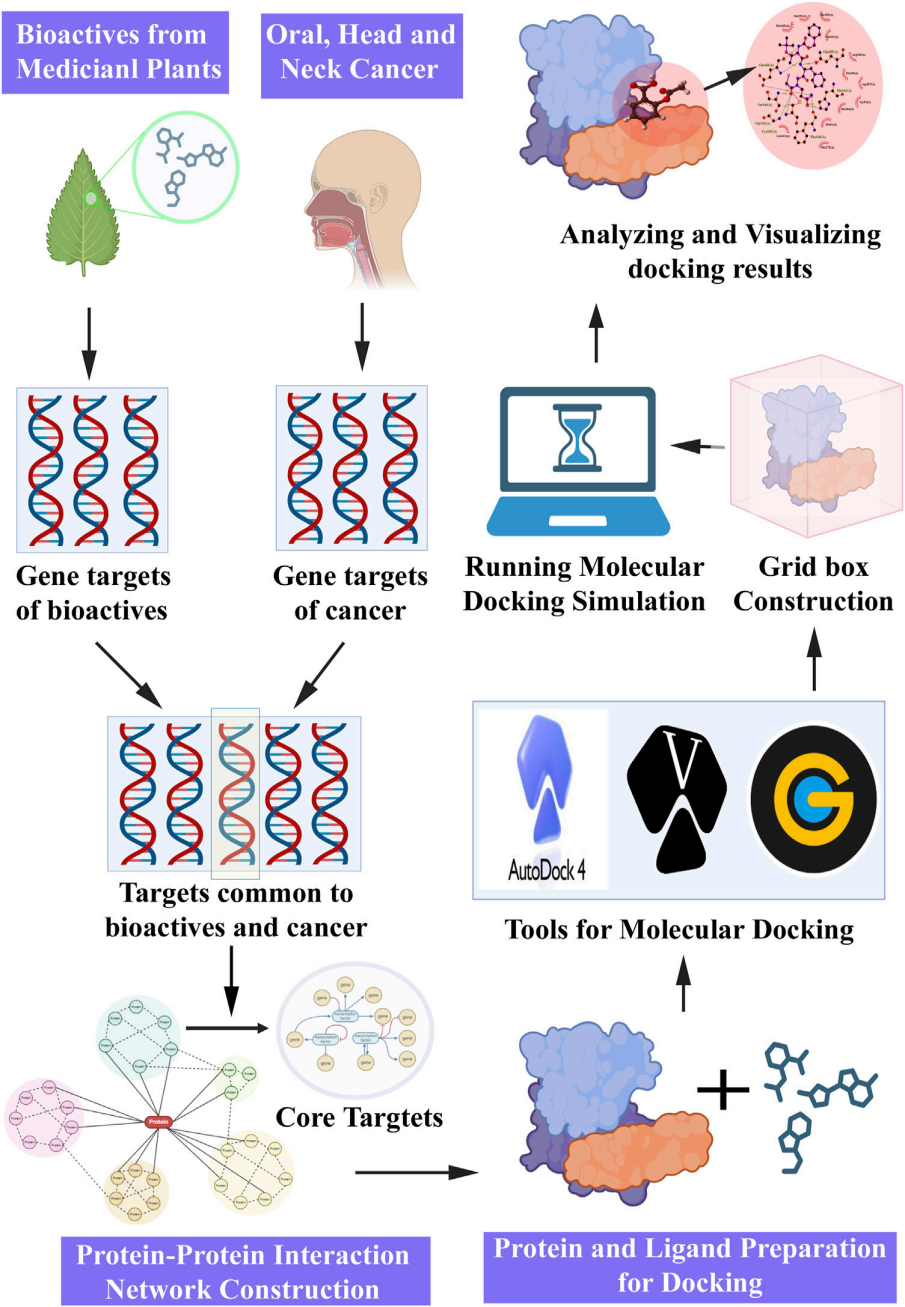
Steps involved in network pharmacology and molecular docking approach for pharmacological activities.

Molecular docking is a computational approach extensively employed in drug discovery and development, and is often complemented by NP. The integration of molecular docking simulations with NP enables the prediction of interactions between potential bioactive molecules and specific protein targets involved in cancer pathways. The sequential steps involved in the molecular docking experiment proceeding with the NP study (Figure 4) were as follows.

### 5.1 Preparation of the ligand

Ensuring an optimized geometry of the ligand is imperative for successful molecular docking simulations. The 3D structure of ligands can be obtained from chemical databases, namely, PubChem (https://pubchem.ncbi.nlm.nih.gov/), ChemSpider (http://www.chemspider.com/), ChEMBL (https://www.ebi.ac.uk/chembl/), as well as medicinal plant databases such as IMPPAT (Indian Medicinal Plants, Phytochemistry And Therapeutics, https://cb.imsc.res.in/imppat/) and TCMSP (Traditional Chinese Medicine Systems Pharmacology Database and Analysis Platform, https://tcmsp-e.com/tcmsp.php), where bioactive compounds from medicinal plants are listed (Ru et al., 2014; Mohanraj et al., 2018). In the case of a lack of ligand structure files, molecular modeling tools such as ChemSketch (https://www.acdlabs.com/resources/free-chemistry-software-apps/chemsketch-freeware/), ChemDraw (https://revvitysignals.com/products/research/chemdraw), and OpenBabel (https://openbabel.org/) can be utilized to generate the necessary 3D structure files. Once the 3D structure file is procured, preparation steps are undertaken, including assignment of partial charges and protonation states, generation of tautomers, addition of missing atoms, removal of water and nonessential groups, and geometry optimization (energy minimization) (Madhavi Sastry et al., 2013).

### 5.2 Preparation of protein

The 3D structure of the protein target, identified through an NP study, can be sourced from protein structure databases, such as the Protein Data Bank (PDB), a comprehensive repository of protein structures determined through techniques such as X-ray crystallography, nuclear magnetic resonance (NMR), and electron microscopy (https://www.rcsb.org/). Alternatively, if the structure is not available in the database, 3D protein structures can be generated using homology modeling or *ab initio* methods based on the protein sequence. Subsequently, essential protein preparation steps must be executed, including the removal of water molecules and other heteroatoms, addition of hydrogen atoms, assignment of partial charges, and protonation states to amino acids, followed by energy minimization (Tripathi and Misra, 2017).

### 5.3 Docking parameters and simulations

After preparing the ligand and protein files, it was essential to configure the required parameters for conducting the docking simulation. Molecular docking can be executed either in site-specific mode, where a 3D grid is centered around a specific binding site, or in blind mode, where the grid encompasses the entire protein structure. The grid defines the spatial coordinates used to evaluate ligand positions during docking. Molecular docking tools, such as Autodock (https://autodock.scripps.edu/) and Autodock Vina (https://vina.scripps.edu/), require the user to set parameters including ligand and protein flexibility, search algorithm, number of runs, population size, number of generations, and scoring function prior to initiating the docking simulation. Other widely used molecular docking tools, such as DOCK (https://dock.compbio.ucsf.edu/), Glide (https://www.schrodinger.com/products/glide), and Gold (https://www.ccdc.cam.ac.uk/solutions/software/gold/), also offer a range of customizable parameters. With the correct configuration of docking parameters, the ligand and protein underwent molecular docking simulations, providing valuable insights into their potential interactions.

### 5.4 Analysis and visualization of docking results

The docking results were subjected to comprehensive analysis, primarily focusing on the binding energies generated during the docking process, where the ligand was docked onto various regions of the protein. The pose, representing the specific conformation of the ligand docked onto a particular region of the protein, was assessed based on its binding energy, which serves as a key indicator of favorable interactions and strong binding affinity. Consequently, the pose with the lowest binding energy was assigned the highest ranking. Following the examination of binding energies for diverse poses, the highest-ranking pose was visually inspected using specialized visualization tools such as PyMOL (https://pymol.org/2/), RasMol (https://www.openrasmol.org/), and Discovery Studio Visualizer (https://discover.3ds.com/discovery-studio-visualizer-download). Visualization serves as a valuable aid in comprehending the binding modes and identifying potential key residues that actively participate in ligand–protein interactions.

### 5.5 Molecular docking of bioactives against oral cancer targets

Recently, molecular docking has become a prevalent technique employed either independently or in conjunction with NP to assess the affinity of bioactive compounds toward oral cancer targets. For instance, Jayaraman et al. (2022) conducted a molecular docking investigation of bioactive constituents extracted from *Eclipta alba,* targeting the oral cancer proteins AKT1 and AKT2 using the Autodock tool. AKT1 and AKT2 are crucial for promoting cancer cell survival by conferring resistance to apoptosis (Degan and Gelman, 2021). The bioactive compounds subjected to docking simulations encompass stigmasta-3, 5-dien-7-one, 9, 12-octadecadienoic acid, and palmitic acid. Among these bioactive compounds, palmitic acid exhibited a high binding affinity towards AKT1, displaying a binding energy of −5.88 kcal/mol. The interaction between palmitic acid and AKT1 involves the formation of hydrogen bonds with the residues THR160 and PHE161. Similarly, 9, 12-octadecadienoic acid demonstrated binding to Akt2 with a binding energy of −5.65 kcal/mol and engaged in forming two hydrogen bonds with the LYS160 residue.

On the other hand, Yu et al. (2022) conducted a study utilizing NP in conjunction with molecular docking to validate the observed results. The bioactive compounds present in the citrous peel were subjected to molecular docking simulations against the core targets predicted by the NP. Notably, the compounds sitosterol, 5,7-dihydroxy-2-(3-hydroxy-4-methoxyphenyl)chroman-4-one, and naringin demonstrated the most substantial binding affinities towards the targets CDK1 (−9.6 kcal/mol), ESR1 (−8.4 kcal/mol), SRC (−9.6 kcal/mol), and PIK3R1 (−8.7 kcal/mol), respectively. Furthermore, sitosterol, hesperidin, and hesperidin exhibited equal binding affinities (−6.7 kcal/mol). Similarly, Hou et al. (2021) employed NP to elucidate the potential oral cancer targets of bioactive compounds derived from *Scutellaria baicalensis* Georgi. Through network analysis, five core targets (VEGFA, MAPK3, AKT1, SRC, and PIK3R1) and nine major hub compounds were identified. Subsequently, molecular docking simulations were conducted with the core targets and hub compounds, leading to the observation that baicalein exhibited a favorable binding energy of < −7 kcal/mol for all investigated targets and established four hydrogen bonds with VEGFA.

### 5.6 Molecular docking of bioactives against HNC targets

Molecular docking is a crucial and widely utilized approach for the preliminary screening and selection of potential drug candidates targeting HNC. Top-ranking compounds identified through docking studies are often subjected to rigorous *in vitro* and *in vivo* assays. A study by Lv et al. (2020) presented a comprehensive evaluation of piperlongumine (PL) as a candidate agent against HNC using *in silico*, *in vitro*, and *in vivo* experiments. Through *in silico* studies, AKT1 emerged as a promising gene target for the treatment of head and neck cancer. Subsequent docking simulations demonstrated a favorable binding interaction between PL and AKT1, with a binding energy of −4.69 kcal/mol. The binding interface involved specific interactions with negatively charged residues of the target protein. *In vitro* analysis further corroborated the high binding affinity of PL towards AKT1, as evidenced by an equilibrium dissociation constant of 123.3 μM. These findings reinforce the potential of PL as a viable therapeutic candidate for HNC treatment. An experiment using FaDu cell-transplanted mice was conducted to validate the *in vivo* efficacy of the PL. Administration of PL to mice led to a significant reduction in tumor size compared to the control group, indicating its potential anti-tumor activity *in vivo.* Gao et al. (2021) employed a molecular docking approach to elucidate the mechanism underlying the inhibition of LSCC proliferation by the Baiying Qinghou decoction. The findings demonstrated that the active constituent quercetin exhibited a notably higher binding affinity towards one of the targets, NO3 (−7.12 kcal/mol). Furthermore, Ling et al., 2022 reported a significant binding affinity of the bioactive compound celastrol towards nasopharyngeal carcinoma (NPC) targets, namely, IL6, VEGFA, and TNF. Additionally, NPC cells treated with celastrol exhibited reduced expression of VEGFA and TNF-. Collectively, these studies provide compelling evidence that the molecular docking approach, when combined with NP, effectively confirms the anti-head and neck cancer properties of bioactive compounds.

## 6 Discussion

NP is a modern approach and utilizes “multicomponent-multitarget” complex network model in drug development (Hopkins, 2007). Numerous drugs act through several targets. This method provides a detailed, in-depth, and comprehensive study of drug-disease interactions and is utilized to determine the mechanistic role of natural products (Yu et al., 2020; Li et al., 2021). NP have been widely employed to explore drugs against numerous diseases, including cancer (Jeyasri et al., 2020; Muthuramalingam et al., 2020; Aarthy et al., 2022; Adarshan et al., 2022; Muthuramalingam et al., 2023a; Muthamil et al., 2024). However, the implication of NP in drug development against from natural sources oral and HN cancer has recently emerged due to the post-treatment emergence of oral cancer and the adverse effects of synthetic drugs.

Similar to other diseases, NP could contribute to the exploration of drug molecules against multiple targets in oral and HNC. NP identifies crucial target genes and pathways implicated in tumor formation and progression by combining diverse data sources, such as genomics, transcriptomics, proteomics, and metabolomics. The potency and toxicity of drug molecules can also be predicted using the NP approach. In addition, NP governs a system-level understanding of drug interplay with targets, which is key for producing safer and more powerful anticancer therapies.

## 7 Limitations

NP is primarily involved in drug development, which helps revitalize natural medicines. However, the bottlenecks in implying NP research on natural medication will be solved in the future. Numerous publicly available databases are essential for NP studies to determine the active ingredients and disease-specific targets. Apart from their curation, inconsistencies in databases are caused by a diversity of information sources, theories, and experimental outcomes. Furthermore, the pharmacokinetic features of drug constituents are determined by the ADMET approach in NP, which mandates experimental validation. The dissection of putative targets relies on the one or often one single database, due to the less numbers of publicly available databases. Compilation of diverse databases into a comprehensive database will be useful for improving the potential of NP studies. Further experimental validation is necessary to confirm the results obtained via NP analysis.

## 8 Conclusion

NP is a powerful approach to unravel the intricate molecular mechanisms underlying various diseases. In addition, natural compounds, such as parthenolide, and traditional Chinese medicine, such as YWLS, have the potential to treat these cancers. This review discusses a diverse range of methods, from metabolomics and lipidomics to NP and molecular docking and provides comprehensive insights into potential treatments and therapeutic targets. The pursuit of innovative strategies offers hope for novel avenues in cancer therapy. Publicly accessible databases play a pivotal role in enhancing our understanding of these diseases. Additionally, it offers a treasure trove of information on genes, miRNAs, drugs, and genetic variations related to oral, head, and neck cancer. Databases, such as HNOCDB, HNCDB, OrCGDB, OrCa-dB, and dbGENVOC, are crucial resources for researchers and clinicians. They facilitate precision medicine research and provide a platform for the integration of genomic technologies including transcriptomics, gene sequencing, proteomics, and microarrays, which have generated extensive data in this field. Furthermore, the field of NP has benefitted significantly from the availability of various databases and tools. These encompass databases related to traditional Chinese medicine, compound and drug information, target interactions, and gene-disease associations. Additionally, advanced algorithms, such as Random Walk and PRINCE, along with visualization tools, such as Cytoscape and Pajek, enable researchers to analyze and understand complex molecular networks more comprehensively. These integrated approaches hold great promise for advancing our knowledge of these diseases and developing more effective and personalized treatment strategies. By sharing data and collaborating and continuously evolving these resources, the scientific and medical community can drive significant progress in the fight against complex and challenging cancers. To achieve significant medicinal advancemnets in the future, NP must be combined with cutting-edge, real-time research. Overall, this review lays the touchstone for further exploration of traditional medicines, their protective mechanisms in oral, HNC treatments, and the applications of NP in drug discovery.
